# Family and community resilience: a Photovoice study

**DOI:** 10.1186/s12939-024-02142-2

**Published:** 2024-03-19

**Authors:** Yvonne Tan, Danielle Pinder, Imaan Bayoumi, Rifaa Carter, Michele Cole, Logan Jackson, Autumn Watson, Bruce Knox, Sophy Chan-Nguyen, Meghan Ford, Colleen M. Davison, Susan A. Bartels, Eva Purkey

**Affiliations:** 1 School of Medicine, Queen’s University, 80 Barrie Street, Kingston, ON K7L 3N6 Canada; 2 Department of Public Health Sciences, Queen’s University, 62 Fifth Field Company Lane, Kingston, ON K7L 3N6 Canada; 3 Department of Family Medicine, Queen’s University, 220 Bagot Street, Kingston, ON K7L 5E9 Canada; 4 Department of Psychology, Queen’s University, Humphrey Hall, 62 Arch Street, Kingston, ON K7L 3N6 Canada; 5 Department of Emergency Medicine, Queen’s University, 76 Stuart Street, Kingston, ON K7L 4V7 Canada

**Keywords:** Photovoice, Resilience, Adverse childhood experiences, Adverse community environments, Participatory action research, Health equity

## Abstract

**Background:**

Adverse childhood experiences (ACEs), in combination with adverse community environments, can result in traumatic stress reactions, increasing a person’s risk for chronic physical and mental health conditions. Family resilience refers to the ability of families to withstand and rebound from adversity; it involves coping with disruptions as well as positive growth in the face of sudden or challenging life events, trauma, or adversities. This study aimed to identify factors contributing to family and community resilience from the perspective of families who self-identified as having a history of adversity and being resilient during the COVID-19 pandemic.

**Methods:**

This study used Photovoice, a visual participatory research method which asks participants to take photographs to illustrate their responses to a research question. Participants consisted of a maximum variation sample of families who demonstrated family level resilience in the context of the pair of ACEs during the COVID-19 pandemic. Family members were asked to collect approximately five images or videos that illustrated the facilitators and barriers to well-being for their family in their community. Semi-structured in-depth interviews were conducted using the SHOWeD framework to allow participants to share and elucidate the meaning of their photos. Using thematic analysis, two researchers then independently completed line-by-line coding of interview transcripts before collaborating to develop consensus regarding key themes and interpretations.

**Results:**

Nine families were enrolled in the study. We identified five main themes that enhanced family resilience: (1) social support networks; (2) factors fostering children's development; (3) access and connection to nature; (4) having a space of one’s own; and (5) access to social services and community resources.

**Conclusions:**

In the context of additional stresses related to the COVID-19 pandemic, resilient behaviours and strategies for families were identified. The creation or development of networks of intra- and inter-community bonds; the promotion of accessible parenting, housing, and other social services; and the conservation and expansion of natural environments may support resilience and health.

## Background

Adverse childhood experiences (ACEs), in combination with adverse community environments, can result in traumatic stress reactions, increasing a person’s risk for chronic physical and mental health conditions [1–12].

### Adverse childhood experiences

The capacity for individuals to reach their full potential is linked to early life experiences. The original study on the effect of ACEs on adult health conducted in 1988 surveyed patients on various stressful experiences in childhood [1]. The ACEs survey questions focused on experiences of physical, sexual, and psychological abuse or neglect, household members with substance use disorders, mental illness, suicidality, or history of incarceration, and exposure to domestic violence [1]. The study found a strong correlation between ACEs and many mental and physical health outcomes, physiological indicators of future health status, and premature mortality—compared to those who had experienced no ACEs, individuals who had experienced four or more categories of childhood adversity had a 4- to 12-fold increase in risk for alcoholism, drug abuse, depression, and suicide attempts [1]. Additionally, adverse childhood experiences were associated with an increased risk of diseases such as ischemic heart disease, cancer, chronic lung disease, skeletal fractures, and liver disease [1].

Numerous subsequent studies have likewise found relationships between ACEs and later health outcomes. For example, experiencing ACEs has been associated with decreased life expectancy [2], homelessness at an earlier age [3], more prescription medications [4], and high healthcare utilization [5].

The impact of ACEs has been found to be compounded with social determinants of health — in other words, the conditions in which people are born, grow, work, live, and age that influence health outcomes [13]. For example, the prevalence of ACEs has been found to be higher in racialized populations [14, 15]. Furthermore, ACE scores tend to be disproportionately high in people experiencing homelessness [16] and previously incarcerated individuals [17]. These same populations are often impacted by social determinants of health such as experiences of racism, poor access to housing and food, poverty, and inequitable education and health care [6, 15, 18].

Addressing ACEs, particularly in communities that are differentially marginalized by social determinants of health, can increase population-level wellbeing and longevity as well as prevent trauma-related outcomes and coping strategies, ultimately helping promote thriving children, families, and communities [6].

### Adverse community environments

Adverse community environments are also closely correlated with decreased wellness. Poverty, for example, is related to markedly worse child and youth physical, mental and developmental health as well as increased mortality rates from a range of diseases [7, 8, 12]. Additionally, poor housing affordability and quality, residential stability, and neighborhood opportunity are associated with multiplicative deleterious health effects [9]. Exposure to community violence is associated with increased aggressive behaviour and depression [10], while experiences of community disaster trauma are associated with poor mental health, including depression and anxiety as well as substance use and disruptive behaviours [11].

### Impact of the COVID-19 pandemic

Increased exposure to ACEs—for example, through intimate partner violence, child maltreatment, substance use, social and emotional trauma [19–23]—as well as increased awareness of racialized violence [24] and public health countermeasures during the COVID-19 pandemic [23, 25, 26] have resulted in substantial impacts on the wellbeing of children and families. Emerging research suggests that certain groups already affected by the pair of ACEs have been disproportionately impacted by the COVID-19 pandemic [27–30]. These impacts include unaddressed increased rates of social isolation, parent and child psychological concerns, as well as increased academic, social and developmental impacts [31]. These may have important implications for the long-term wellbeing of communities, emphasizing the importance of early intervention.

### Family resilience

Despite experiencing adversity, supports can be reinforced for vulnerable children, families, and communities so that together they may thrive. In order to better understand how to address needs of individuals and communities impacted by the effects of ACEs, it is important to consider the construct of resilience.

The majority of research on psychosocial resilience has focused on individual resilience. For example, studies of youth who have thrived despite parental dysfunction have focused largely on extrafamilial resources, including mentorship from coaches and teachers [32]. Additionally, there is an assumption that families contribute to risk, but not to resilience [32]. However, adverse environments have an impact on the whole family, and key family processes can mediate adaptation for all individual members, their relationships, and the family unit.

Family resilience refers to the ability of families to withstand and rebound from adversity; it involves coping with disruptions as well as positive growth in the face of sudden or challenging life events, trauma, or adversities [33]. One of the leading frameworks on family resilience is the Walsh Family Resilience Framework, which organizes family resilience into three key processes: shared beliefs (seeing things in the same way or having a similar positive outlook), organizational processes (adaptability, familial connectedness, access to social and economic resources), and communication/problem solving processes (emotional information sharing and open communication, collaborative problem-solving) [32]. Although some families may be more vulnerable to or face more hardships than others, a family resilience perspective is grounded in the belief that families can overcome adversity and grow over the life course and across generations.

### Community resilience

Community resilience has also increasingly been recognized as an important tool to address adversity and build stronger communities to support health and wellbeing. Community resilience is the capacity to anticipate risk, limit effects, and recover rapidly through survival, adaptability, evolution, and growth in the face of turbulent change and stress [34, 35]. At its core, community resilience is supported by equitable, accessible, and sustainable systems; it involves building capacity to adapt to disruption, in positive ways, and acquiring the necessary resources to support community members during the disruption [36, 37].

## Methods

### Aim

This study aimed to identify factors contributing to family and community resilience from the perspective of families who self-identified as having a history of adversity and being resilient during the COVID-19 pandemic.

### Design

We conducted a community based participatory research (CBPR) project which was guided by critical theory. CBPR is a tool for democratic engagement, enhancing community resilience and agency through the process of co-creation [38]. It is an iterative approach to knowledge generation that recognizes collaboration as core to meaningful knowledge production and involves community members in all stages of the knowledge production process, from identification of research questions through to data collection, interpretation, and knowledge translation. Critical theory identifies unequal distribution of power and resources as central to existing inequities in wellbeing [39]. Using CBPR guided by critical theory allows for the meaningful elevation of lived experiences, thereby disrupting traditional research hierarchies to focus on the expertise of participants in the process of co-creation, particularly as it relates to addressing inequities in wellbeing [38].

This study used Photovoice, a visual participatory research method which asks participants to take photographs in response to a research question [40]. Photovoice enables individuals to reflect on community strengths and barriers, to promote critical dialogue through group discussion of photographs, and to reach policymakers. Photovoice has been adopted by researchers to explore the needs of different populations, such as people with physical and intellectual disabilities [41, 42], people who use substances [43], and people experiencing homelessness [44].

### Setting and participants

This study consisted of a maximum variation sample of families who demonstrated family level resilience in the context of the pair of ACEs during the COVID-19 pandemic. This was part of a larger study: “Engaging Families to Build Healthy Communities” which involved both qualitative and arts-based methods to understand the experiences of families with history of adversity who self-identified as being resilient during the COVID-19 pandemic.

Working with partner organizations, participants were invited to participate if they were 1) families, defined as one or more adults living with and acting as primary caregivers for one or more children under the age of 18; 2) who self-identified as having experienced adversity, including adverse childhood experiences or adverse community environments; 3) who were residents of the Kingston, Frontenac, Lennox, & Addington (KFL&A) region; and 4) who could consent for all family members to participate in all components of the study. The use of maximum variation considered factors such as household composition (single parent, two parent household, ages of children), ethnocultural background (newcomer, Indigenous, racialized), geographic location (urban vs. rural) within KFL&A, among other factors. The goal of these case studies was not to be representative of the local population, but rather to identify cases that reflected diverse community experiences and that can inform interventions relevant to other families experiencing adversity.

### Data collection and analysis

Prior to the study, ethics approval was obtained from the Health Sciences Research Ethics Board (HSREB) at Queen’s University. Verbal consent was obtained from participants, and voluntary participation and confidentiality were ensured.

Family members were asked to use a cellphone or tablet (which was provided if needed) to collect approximately five images or videos that illustrated the facilitators and barriers to well-being for their family in their community. Family members could engage in this activity together or individually and were given three weeks to collect images.

Semi-structured in-depth interviews were conducted using the SHOWeD framework to allow participants to share and elucidate the meaning of their photos [45]. Discussions utilized a phenomenological, exploratory approach to learn more about the specific experiences of families as well as the effects the pandemic has had on their lives, social and mental health, and what has contributed to their ability to be resilient. Each interview was audio recorded and transcribed verbatim and translated into English if needed. Through the use of NVivo software, two researchers then independently completed line-by-line coding using thematic analysis, an interpretative approach to qualitative data analysis that enables the identification and interpretation of patterns of meaning within data sets [46]. The process of thematic analysis involved (1) dataset familiarisation; (2) data coding; (3) initial theme generation; (4) theme development and review; (5) theme refining, defining and naming; and (6) writing [46].

## Results

Nine families were enrolled in the study. Families ranged from two to five members and included single parent and two parent families. There was representation from Indigenous, Muslim, newcomer, military, and francophone families. Family members included individuals identifying as 2SLGBTQ + , individuals with a history of substance use, individuals who have experienced homelessness, survivors of sexual violence, individuals living with chronic illnesses, and individuals with mental health issues.

Although the stories and photos that families produced were diverse and covered a wide range of topics, five main themes were identified from the data analysis to reveal factors that enhanced family resilience: (1) social support networks; (2) factors fostering children's development; (3) access and connection to nature; (4) having a space of one’s own; and (5) access to social services and community resources.

### Theme 1: Social support networks

Participants illustrated the importance of social support networks in their local community in helping them navigate difficult situations and circumstances. For example, many participants were able to find people similar to them, allowing for a sense of belonging, connection, and support. Finding like-minded individuals who shared interests, values, and aspirations with participants was highlighted as crucial to fostering an environment where families could openly express themselves and feel understood. For one mother, a community centre for mature female-identifying university students offered a space for her to connect with others who, unlike many other university students, also had children. In describing a photo of the centre, she expressed how she was able to receive support and understanding:

*“I would say most important is the friendship from the mature students. And we resonate [with one another’s] struggles. The struggles of balancing study and family life, especially time with our children.”* (Family 043)

Another family noted that their local religious community provided them with a sense of inclusion. Over the pandemic, they partook in religious practices at home, but were unable to gather with others; being part of their local mosque allowed them to both feel more connected to their community and strengthen their faith and spiritual growth:

*“My husband who regularly goes over [to the local Mosque] [...] feels like he belongs, really since, they pray together.”* (Family 001)

While discussing a photo of a local park that was important in helping build her family’s resilience, one parent described it as a space where she was able to meet others who were in similar circumstances as her. These networks allowed her to feel a sense of camaraderie and togetherness, which in turn enabled her family to withstand and overcome barriers:

*“The people who are going to the park [...] have kids the same age as you, live in kind of the same lifestyle or at least live in the same neighborhood. So it’s a good kind of common meeting ground for people. That’s where we’ve made some connections in the community and obviously that’s important for resilience”* (Family 009)

**Fig. 1:**
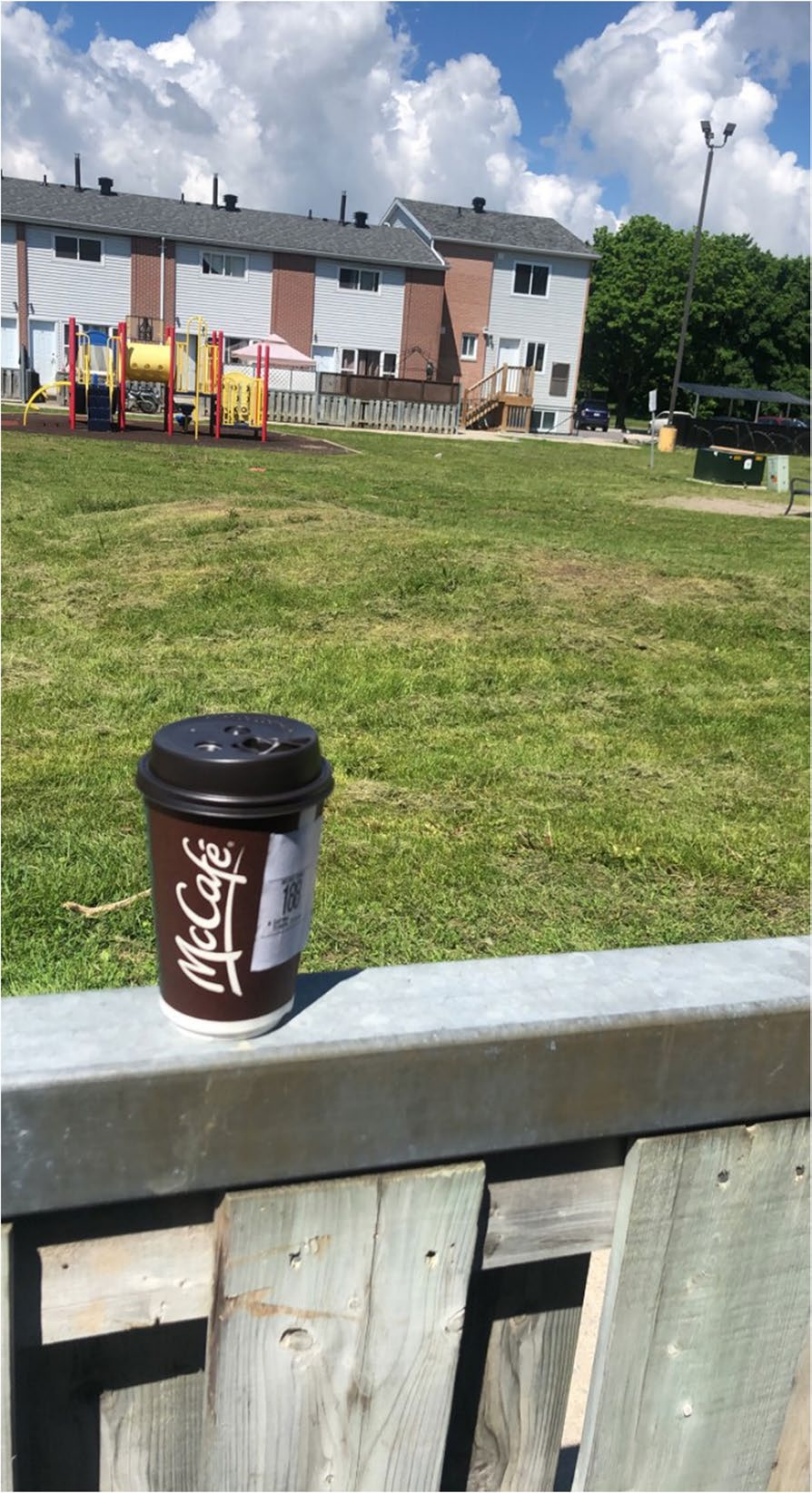
Social support networks

Families also noted that fostering bonds through the exchange of assistance, resources, and goodwill not only helped address immediate needs, but also nurtured a sense of belonging and shared responsibility. Creating such networks of mutual aid and collaboration was highlighted as helping lower barriers faced by families and develop their resilience. For Family 001, the relationship her family built with their babysitter – described as mother-daughter relationship – was essential; the babysitter gave the family everything from advice to food, providing a person for the family to rely on in emergencies. Having someone in the community to rely on helped alleviate the family’s burdens as well as promote interconnectedness. Similarly, Family 007 had a friend deliver food during times of hardship. The COVID-19 pandemic limited her capacity, both mental and physical, to leave the house and purchase food; knowing that their family was cared for by others in the community was highlighted as a source of resilience and strength. Family 003 took a photo of coffee their neighbour had brought them one morning (Fig. 1), and shared that their neighbours were always willing to offer help with childcare when needed. This ability to look out for one another, and in particular, the respect and honesty associated with it, was noted as a source of security for the family:

*“I know if I was to run inside while my sons outside, he’d be 100% taken [care of] and looked after by people in the neighbourhood.”* (Family 003)

Such community reciprocity also emerged as a source of resilience for Family 047. Being able to both help others and receive help was important in helping foster a sense of interdependence and cultivate a culture of reciprocity, ultimately enhancing the overall well-being of the entire community:

*“It’s just people, community, sharing. I have this [...] coffee table that I don’t need anymore. Does anybody need this? Or I have a bag full of single bed bedding, does anybody need this?”* (Family 047)

### Theme 2: Factors fostering children’s development

Participants consistently acknowledged the importance of caring for their children in helping them to overcome adverse circumstances and stressors.

Many families focused on the importance of play. Public spaces for play in the community were noted to foster social interactions, teaching children essential interpersonal skills such as cooperation and communication as well as developing friendships. Play was also highlighted as a way for family members of all ages to spend time together and engage in genuine face-to-face interactions, cultivating a sense of unity and togetherness. One parent expressed that playing board games was an important family activity that contributed to their resilience:

*“I really enjoy board games and I shared my love of it with the kids and they enjoy it as well. And it’s a way for us to get together.”* (Family 042)

Free play was also highlighted as a way for children to explore, learn, and challenge themselves – in addition to physical benefits, a safe environment to play was noted to instill a sense of confidence, encouraging children to take risks and engage in creative, unstructured diversion. In describing the playground as a source of strength for her family, one parent shared that:

*“It’s like a safe spot where she can go and play that’s age appropriate. She can meet her friends and then she can just [...] engage in free play which is, I think, really important. Like so much of their day is structured and this just gives them the opportunity to just go out and be kids.”* (Family 009)

**Fig. 2:**
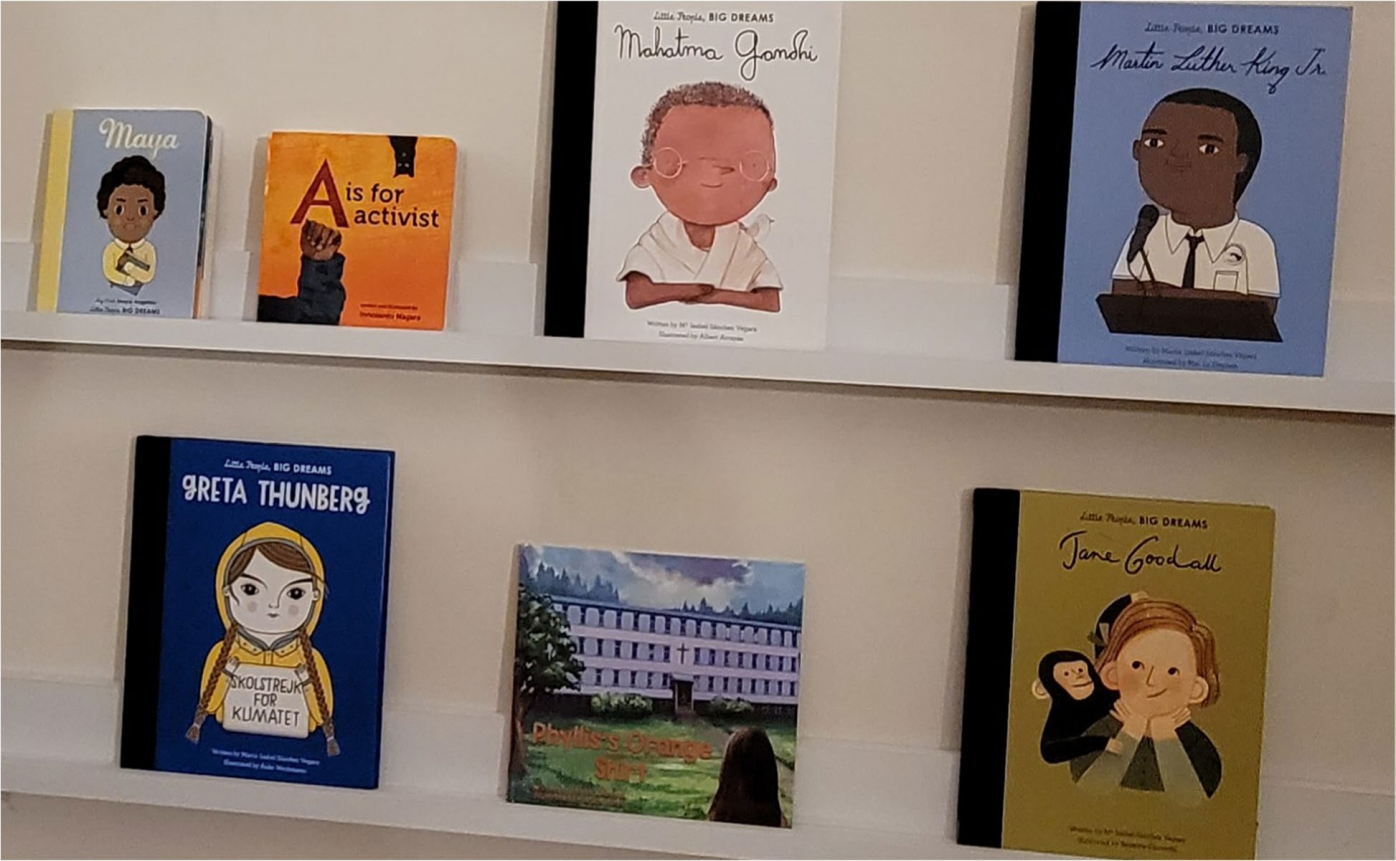
Factors fostering children’s development

Creating opportunities for children's education was also highly valued. Learning opportunities were highlighted as providing children with skills and values crucial for their personal and intellectual development – many families mentioned that through education, children were equipped with tools to explore their interests, discover their passions, and realize their potential. Families also noted that providing learning opportunities empowered children to become informed and critical thinkers, in addition to nurturing their curiosity and broadening their horizons beyond their immediate surroundings. One parent stated that books were a particularly important resource to do so (Fig. 2):

*“They could be library books or they could be purchased books. They could be digital books. They could be audio books. But the stories are there! We just need to [...] provide these stories to our children so that we are giving them the tools and resources in an age appropriate way for them to learn about things that are going to shape the world.”* (Family 007)

Informal learning opportunities were also important – while formal education provides structure and deliberate teachings, informal education was noted by families as a way for children to develop crucial life skills and foster a deeper understanding of the world around them. For example, many families reflected on the importance of informal interactions with peers, community members, and the environment; these interactions allowed children to be exposed to real-world scenarios that contributed to their holistic development. In describing a photo of a bus stop representing her daughter’s use of public transportation, one parent stated that:

*“It teaches them a lot of life skills which will make them better adults, I would hope.”* (Family 009)

The health and safety of children was another priority for families. Prioritizing children's safety not only ensured the immediate well-being of the youth, but also helped alleviate parental anxieties and strengthen family resilience – several parents noted that having spaces they knew were safe for their children was a source of strength. In describing a photo of a local park, one parent stated that:

*“It’s much safer than the [other local park]. Like I can not let my children go to the [other park] by themselves because it’s an open area and busy. For this park, it’s still an open area, but it’s located in the neighbourhood. So it’s kind of like a neighbourhood park.”* (Family 043)

Likewise, ensuring that children were healthy helped families thrive in the face of adversity. For Family 047, an accurate mental health diagnosis for her daughter not only aided in explaining health concerns, but also ensured that she was receiving the appropriate interventions, treatments, and support tailored to their unique needs:

*“During the pandemic and all of the stops and starts, was when we realized my daughter was struggling a little bit extra. And she was able to get her ADHD diagnoses during that time frame.”* (Family 047)

Allowing children to be independent and to have autonomy was another recurring topic. Families highlighted that fostering children's autonomy was a fundamental aspect of their healthy development and overall well-being. Providing children with the opportunity to make choices and decisions within appropriate boundaries was noted by many families as helping them build a sense of self-confidence, responsibility, and self-reliance. Autonomy also encouraged critical thinking skills by providing children with a chance to evaluate options, make decisions, and understand the consequences of their choices. As aforementioned, public transportation and local coffee shops enabled Family 009 to build their children’s independence:

*“As they get older, they kind of want to have that distance and that sense of autonomy. And having bus passes has given them that, which I think is nice for them. … they’re able to go to [coffee shop] and study and chat and just like be away from their parents.”* (Family 009)

Similarly, for another family describing a photo of their daughter playing in the grass, watching her learn to walk and gain autonomy was a source of strength:

*“It shows that she’s growing, that she is becoming independent per se. She’s giving me the idea of having her own sense of being, her own sense of independence. And she’s learning how to become a little girl.”* (Family 040)

### Theme 3: Access and connection to nature

The ability to access and connect with nature was perceived as a source of resilience for participants. Many noted that time spent in nature proved healing for both their physical and mental health. Nature provided families with space needed to be active and burn off energy. Likewise, nature provided families with an escape from the noise and busyness of life in the city. As a result, its grounding abilities were highlighted extensively by many families, proving to be an invaluable resource for participants’ mental health:

*“I remember during the pandemic […] I could hear the birds louder. And there were no cars driving. And I just felt that connection. I could see the stars a little bit brighter. And I felt this connection to the earth. And I just felt like everything was coming back to life and that the earth was breathing again.”* (Family 007)

**Fig. 3:**
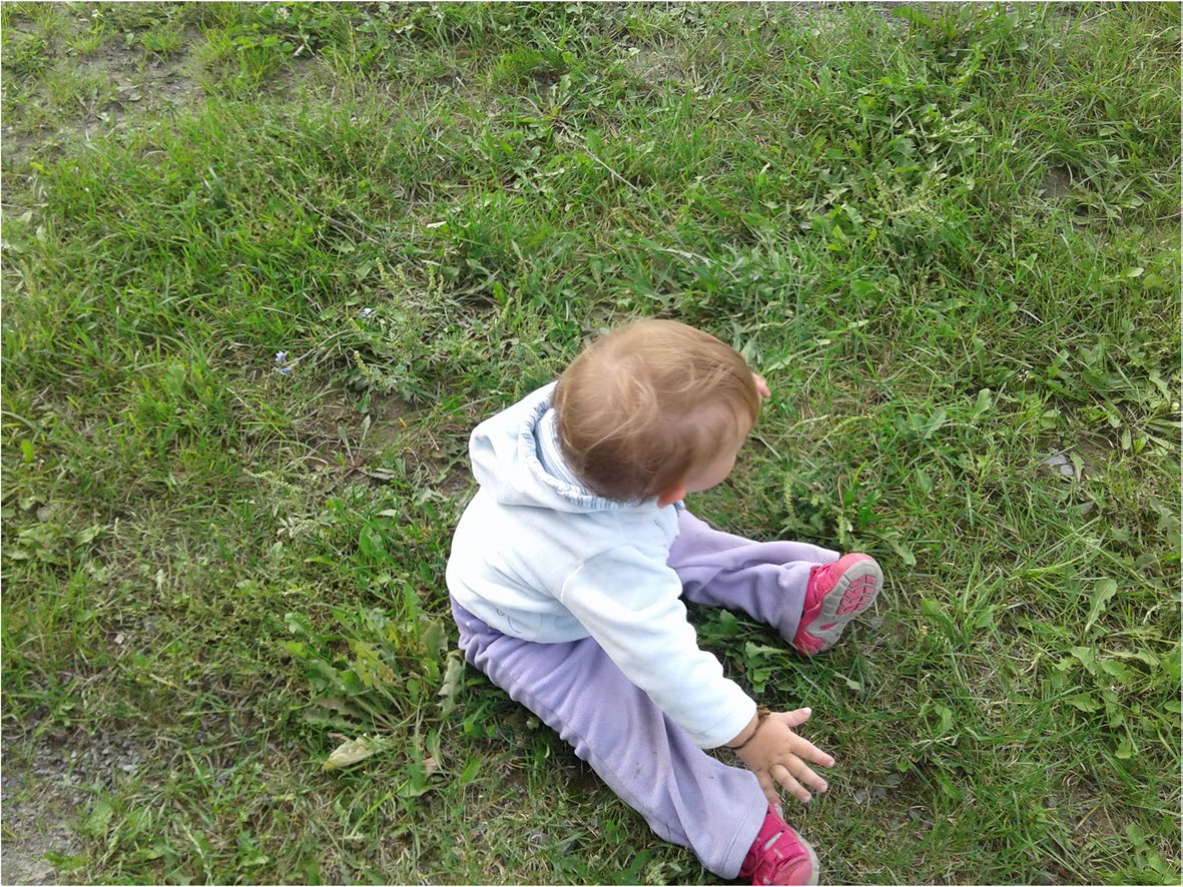
Access and connection to nature

Families noted that nature proved healing for both parents and children alike. Parents highlighted how the outdoors offered a way for their children to expend energy and express themselves as loudly as they desired. Others noted the calming effect that nature had on their children’s demeanor. One family considered moving closer to nature after noticing a drastic change in their daughter’s emotions when spending time in the countryside (Fig. 3):

*“There were a couple [photographs] I took of her sitting on the grass […] She just kept looking up at us once in a while and smiling like it was the best thing in the world […] When she’s in the city she’s absolutely miserable. She doesn’t want to be in the city. She’d rather be out in the country [… ] We’re going to see about going there, to the country, and living there. Because it isn’t only her mental health, it’s our mental health as well […] And I think her mental health is not the greatest right now because she’s not happy.”* (Family 040)

Likewise, families noted that the outdoors allowed for geographically and financially accessible leisure activities. Due to the nature of the pandemic and its various lockdowns, families encountered restrictions in the number of activities they could partake in outside of their homes. Engaging in activities in nature emerged as one of the primary opportunities. While some described activities as simple as playing in their own backyard, others took advantage of the nature trails in their neighbourhood. One family conveyed how engaging with nature was among the few leisure activities available to them during the pandemic:

*“Exposure in nature is good for everybody […] it’s so accessible to everybody. It’s free. It’s easy to get to. When we went into the lockdown and we weren’t allowed to do anything, that [nature] was all that there was. And actually, it was really nice because it forced us to get out there.”* (Family 009)

### Theme 4: Having a space of one’s own

As participants reflected on the challenges encountered during the pandemic, they highlighted having a space of one’s own as a source of strength. For some participants, the home served as an important space that contributed to their resilience. One family in particular reflected on the fact that many lost their homes during the pandemic. Residing in a low-income housing neighbourhood, they stressed that housing held particular significance within their community. Thus, in describing a photo of their living room, the family shared their pride and gratitude for their ability to maintain a home and thrive in it. In doing so, they expressed how ownership and autonomy over their home greatly contributed to their resilience:

*“It was a huge strength for myself and my family that we managed to keep our home and thrive in it […] I made sure that my child is allowed to live in his home […] He's allowed to strip right in the living room, take his shirt off, put it on the counter and I’ll pick it up later. He doesn’t have to do anything right then and there. He’s allowed to live in his home and I am as well […] You can be resilient without a home, but me personally, I needed the home to be resilient.”* (Family 003)

**Fig. 4:**
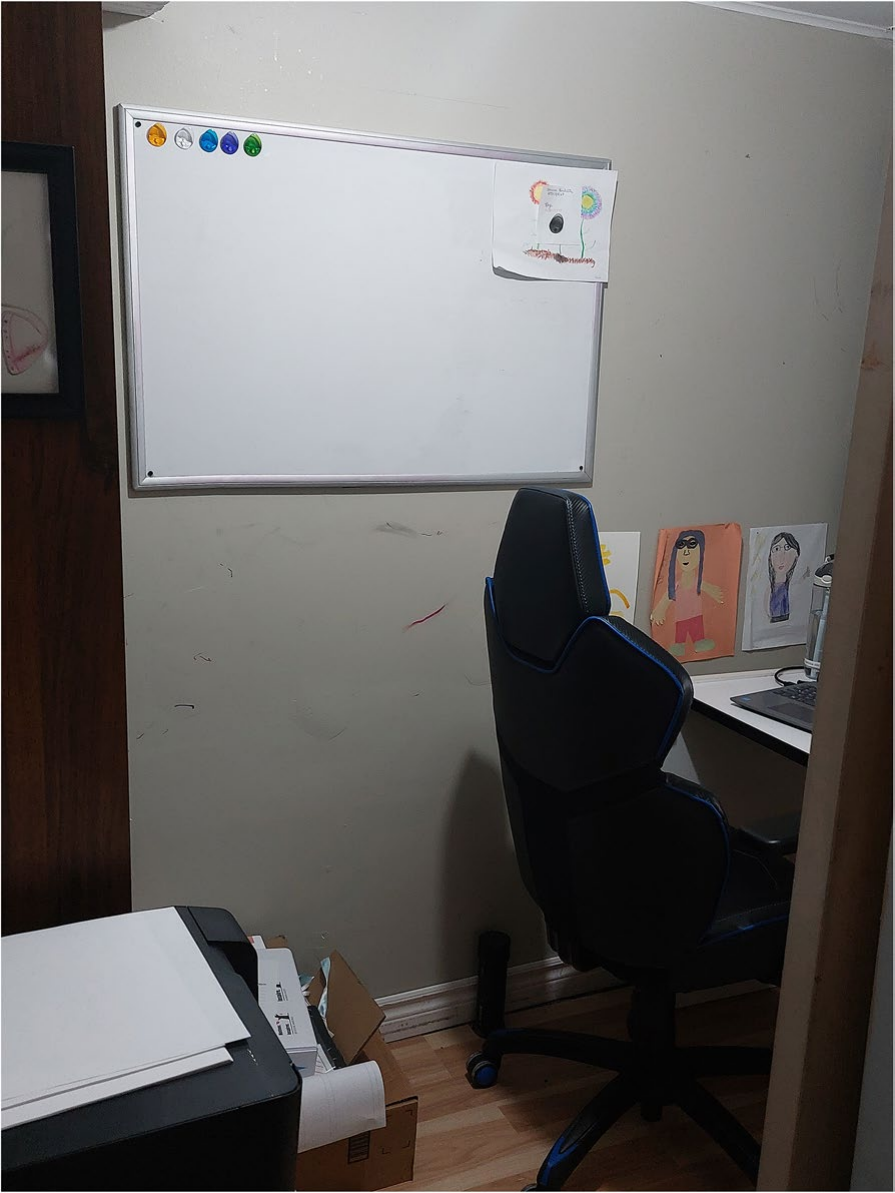
Having a space of one’s own

Other participants noted that having their own space within their homes was essential for their mental well-being. Amidst the different lockdowns, some families grappled with the challenges of close living quarters. Throughout these narratives, participants acknowledged that quality time with their families was appreciated; however, alone time was needed for them to recharge. Thus, having a dedicated space to call one’s own within the household offered a place of sanctuary. This theme stood out in Family 042’s narrative. As the family engaged in homeschooling, they dedicated most of their time to being together. Consequently, the mother noted how her home office provided her valuable solitary moments to recharge and work on personal projects (Fig. 4):

“This [home office] is a really important space for me […] It’s the fact that it’s my space […] I consider myself an extreme extrovert. But even extreme extroverts need their time to just not interact with other people […] I can have things organized the way I want [in this space], and I can work on creative projects […] I feel like if I didn’t have this space to come to and work on things […] I think it would be a lot harder. Because then I would constantly feel like I’m just going to the job, to work for somebody else, and I’m just doing the housework and planning the lessons. And I [wouldn’t] have time to sit down and do something that I want to do that’s just for me.” (Family 042)

### Theme 5: Access to social services and community resources

**Fig. 5:**
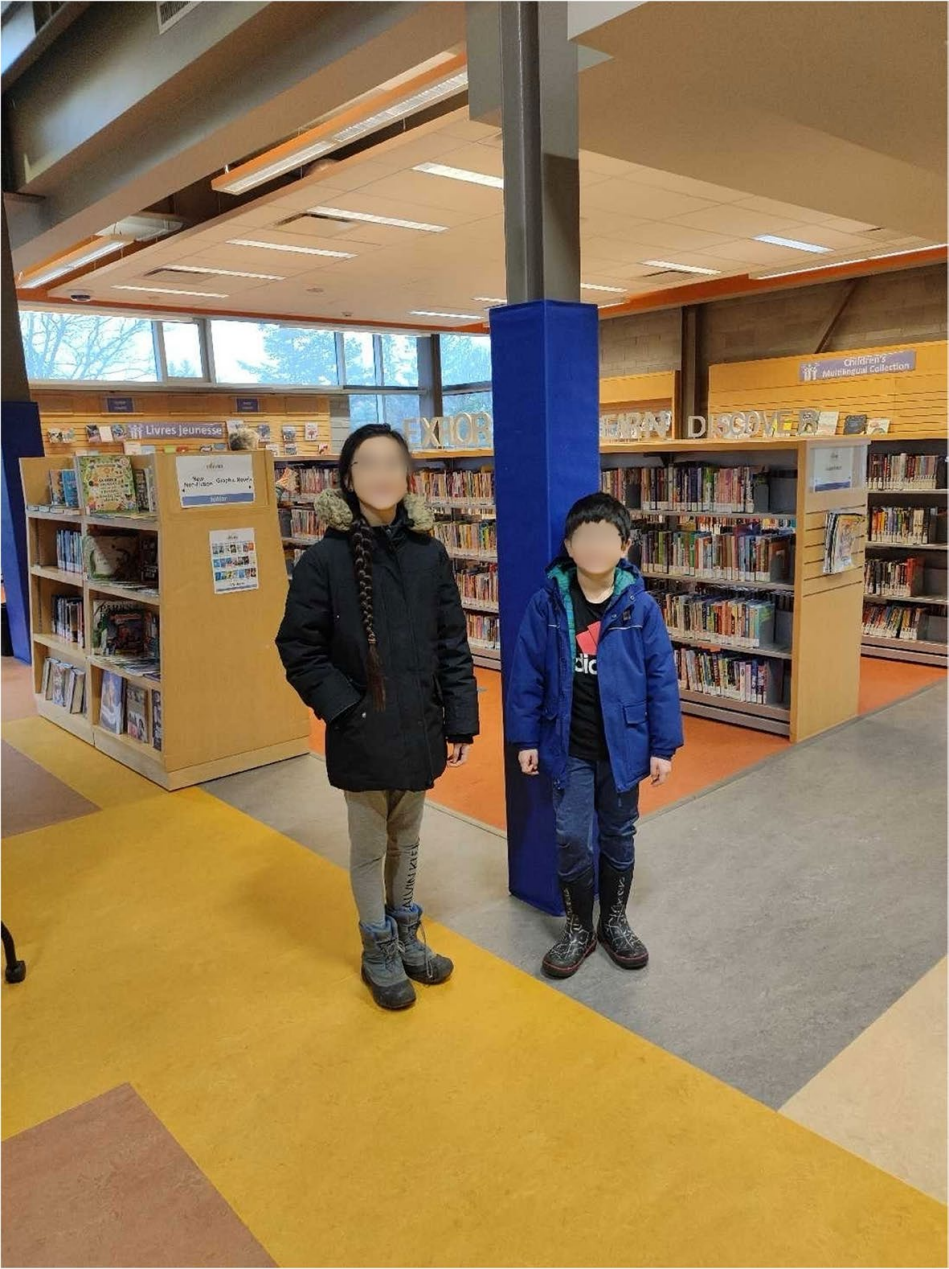
Access to social services and community resources

As participants reflected on the factors that supported their resilience, the ability to access social services and community resources was perceived as a strength. A notable facility for many families was their local library. The library serves as an accessible resource hub for families. They visited the space to access books, in addition to finding entertainment through games and other materials. Others noted that libraries provided them equipment, like printers and scanners, to which they would not have had access otherwise. Notably, the library also provided the families with free WIFI, allowing them to search the internet and complete work. The library was a vital resource for Family 043, who shared that it held particular significance for newcomers like themselves (Fig. 5):

*“For families like us with very limited resources, we don’t have the budget to buy books from the bookstore or, you know, [name of store] all the time. So, the library is really vital […] And not only for entertaining but also for education […] I didn’t grow up in an English-speaking country. And the books that I read may not be suitable for the children here […] I didn’t know how to help my children to get integrated with their schools. And also to learn the culture […] The library is one of the very important resources, and free resources, accessible resources for us as newcomers.”* (Family 043)

Indigenous community resources were recognized for their supportive role. These Indigenous specific programs and services were able to integrate Indigenous ways of knowing and being to support the well-being of participants, while remaining rooted in the importance of cultural identity. In describing a photo of an Indigenous community centre, Family 048 shared how an it provided culturally relevant care and resources that aimed to address their specific needs. They expressed feeling understood and safe in this space, noting how the support they received allowed them to connect with Indigenous culture and identity. Their children were able to play freely in this space, as the centre offered multiple playrooms, toys, and activities. In doing so, this centre created a safe space where the family could access crucial resources and receive assistance in caring for their children:

*“They got like spots where you can get stuff for your kids like diapers, hats or mitts or like pads or like panty liners and tampons […] It’s kind of like a break for us where we can go and like not have to worry about like dealing with the kids. They help with the kids and give us a break to have a coffee and stuff.”* (Family 048)

Likewise, services tailored toward women and children provided families with the resources and care needed to navigate their unique challenges. Family 043 noted that the support they received from a centre for mature, female-identifying students proved invaluable. This centre equipped the single mother and her children with practical support, like financial aid and food, along with non-essential resources like holiday gifts. She described this support as integral to their strength during the pandemic:

*“At the [name of centre], I received the support for not only the financial part but also the emotional and academic part… During the pandemic this centre has provided us many supports and also care. And so this is a very important part of my life and my children’s life.”* (Family 043)

Community Health Centres also stood out as a comprehensive service for health needs. With many resources located in one place, families were able to save both money and time. They were also introduced to other resources through these centres, many of which they were unaware of before visiting. In doing so, these resource hubs provide an effective way of supporting the participants’ needs. One of these resource centres was photographed by Family 047, who commented on its well-designed and impactful nature:

“So for me with the [community health centre], I’m really grateful that it is kind of like a one-stop shop. The dentist is there. My therapist is there. My doctor is there. The [early childhood program] is there. The [other early childhood program] is there now. [Substance use program for mothers] is there now […] The fact that it’s this one-stop shop is hugely helpful for people. Maybe it’s difficult to get to a bunch of different appointments and stuff. And when you have a resource like [community health centre] where it’s all in one […] You can book your kids dentist check-in and doctor check-in in the same day and not have to spend millions in cabs or have to spend three hours on the bus to get them from different place to different place. Like that in itself is just a really smart set up.” (Family 047)

Others highlighted the importance of substance use services as a catalyst for their resilience. These non-judgmental supports provided families with the empowerment needed to improve their mental and physical well-being. Participants highlighted that the consistency and reliability of these services proved critical in their recovery. Overall, these supports served as a driving force in improving the lives of participants. One family shared this sentiment as they discussed the importance of a methadone program in their community:

*“The methadone program in and of itself has been an enormous driving force in my life changing for the better. I’m grateful that it’s there […] When you reach, there’s a hand there, that’s huge resilience. That builds resilience because day after day […] you develop a resiliency in yourself, in being able to potentially trust that that’s going to be there. That there might be more for you in the future. Those things take tremendous resiliency.”* (Family 047)

## Discussion

Five themes were identified from the thematic analysis, reflecting the importance of social support networks, children's development, nature, having a space of one’s own, and access to community resources. Similar to our findings, previous studies have highlighted that families with access to strong support systems, whether through extended family, friends, or communities, are better equipped to navigate and overcome adversities [47–49]. Research has also found that providing both affective and structural support to children can play a pivotal role in shaping and contributing to the overall strength and adaptability of a family unit [48, 50]. Nature has been identified as a key component of the urban health infrastructure, important to supporting resilience and addressing the various health needs of communities [51, 52]. Studies have also shown the importance of stable home environments in providing a sense of physical, emotional, and psychological well-being [47]. A family’s well-being has been found to depend on both how well they access the resources they need to sustain themselves and grow and how well other systems change to meet their needs [48, 50, 53–55].

The fact that the findings of our study, with its emphasis on the voices of traditionally marginalized participants, are consistent with the literature increases the urgency with which these recommendations must be addressed. From both a policy and a community perspective, activities that lead to the creation or development of networks of social support and intra- and inter-community bonds should be prioritized. This may include policies and programs targeted at supporting populations at risk of social exclusion, supporting key life-course transitions, and promoting community development efforts [56]. Creating and funding accessible parenting, housing, and other social services is another way families can be supported [57, 58]. Additionally, given the many benefits to family resilience associated with connections to nature, public health and municipal agencies should work to ensure that natural environments are both conserved and expanded. Furthermore, promoting outdoor play and advocating for green environments in locations that are accessible such as schools and community spaces may support resilience and health [59]. In implementing such policies and programs, it will be important to take a participatory approach and consult the local community, and in particular, work with families facing adversity to develop and build solutions that help them thrive. Centering voices from Indigenous families, newcomer families, single parent families, and families in recovery, for example, would allow for policymakers to better understand the unique barriers and sources of strength of these communities in local contexts.

A strength of the study included the use of community based participatory research, allowing for collaboration, reflection, and mutual learning between stakeholders. By spending substantial time with each family, the study team built trust, and was able to elicit rich information which is often not available from families experiencing adversity. Additionally, the use of Photovoice was a strength as it empowered participants to determine on their own terms the information that they wished to share, and to take an active role in sharing their lived experiences. This method further allows underrepresented voices to meaningfully contribute to academic discourse. A limitation of the study was that the relatively small sample of families.

## Conclusions

Those affected by individual and structural adversity have been especially susceptible to the social and emotional ramifications of the pandemic and ongoing recovery, increasing the urgency of our call to action. The findings of our study reinforce that resilient behaviours, strategies and community structures can serve as protective factors for families in such contexts; however, families and communities will need material and logistical support going forward to implement these healthy and resilient coping strategies. This study used a community based participatory action approach to uncover the factors that contribute to family resilience.

Future studies should look into conducting similar studies across different cities and in families facing different social contexts. Ultimately, factors contributing to family resilience can be leveraged to make tangible changes at the individual, policy, and clinical levels, catalyzing positive changes within the community and enhancing the wellbeing of families.

## Data Availability

No datasets were generated or analysed during the current study.
